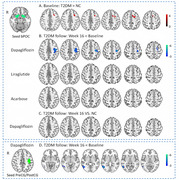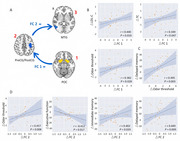# Dapagliflozin restores odor‐induced functional integration of POC circuit, but not local olfactory neural activation in patients with type 2 diabetes: a 16‐week randomized parallel comparative study

**DOI:** 10.1002/alz70859_097012

**Published:** 2025-12-25

**Authors:** Xin Li, Bing Zhang

**Affiliations:** ^1^ Nanjing Drum Tower Hospital, Affiliated Hospital of Medical School, Nanjing University, Nanjing, Jiangsu China

## Abstract

**Background:**

The comparative neuroprotective effects of different hypoglycemic drugs have not been characterized in randomized controlled trials. Here, we investigated the effects of dapagliflozin, liraglutide, or acarbose treatment on the directed functional connectivity of primary olfactory cortex (POC) circuit and local activation under odor stimulation in patients with type 2 diabetes (T2D).

**Method:**

In the 16‐week randomized parallel‐group open‐label trial, 36 patients with T2D, inadequately controlled with metformin, were randomized 1:1:1 to receive dapagliflozin, liraglutide or acarbose. Simultaneously, 36 normal controls were recruited. Olfactory task functional MRI and a battery of olfactory and cognitive tests were conducted in all subjects and postintervention. Generalized psychophysiological interaction analysis was used to identify directed functional connectivity of POC circuit under odor stimulation.

**Result:**

The 16‐week treatment with dapagliflozin restored odor‐induced functional integration of POC‐sensorimotor cortex‐middle temporal cortex circuit with Gaussian random field correction, but liraglutide and acarbose did not, and dapagliflozin tended to improve attention (*P* = 0.071). Liraglutide enhanced odor‐induced activation in the left hippocampus, but dapagliflozin and acarbose did not. The decreased odor‐induced directed functional connectivity was associated with improvements in lipid levels and changes in olfactory threshold, executive function, and memory performance (all P < 0.05).

**Conclusion:**

These results suggest that dapagliflozin and liraglutide have unique neuroprotective effects, respectively. Liraglutide may act on the activation of local olfactory‐related regions, while dapagliflozin acts on the functional integration of neural circuits. These findings highlight the importance of targeting both metabolic and neural pathways in the management of T2D‐related cognitive decline.